# Studying dyadic structure–function relationships: a review of current modeling approaches and new insights into Ca^2+^ (mis)handling

**DOI:** 10.1177/1179546817698602

**Published:** 2017-04-12

**Authors:** Mary M Maleckar, Andrew G Edwards, William E Louch, Glenn T Lines

**Affiliations:** 1 Simula Research Laboratory, Center for Cardiological Innovation and Center for Biomedical Computing, Lysaker, Norway; 2 University of Oslo, Oslo, Norway; 3 Institute for Experimental Medical Research (IEMR), Oslo University Hospital and the University of Oslo, Oslo, Norway

**Keywords:** Electrophysiology, mathematical modeling and simulation, calcium dynamics, excitation-contraction coupling, subcellular modeling

## Abstract

Excitation–contraction coupling in cardiac myocytes requires calcium influx through L-type calcium channels in the sarcolemma, which gates calcium release through sarcoplasmic reticulum ryanodine receptors in a process known as calcium-induced calcium release, producing a myoplasmic calcium transient and enabling cardiomyocyte contraction. The spatio-temporal dynamics of calcium release, buffering, and reuptake into the sarcoplasmic reticulum play a central role in excitation–contraction coupling in both normal and diseased cardiac myocytes. However, further quantitative understanding of these cells’ calcium machinery and the study of mechanisms that underlie both normal cardiac function and calcium-dependent etiologies in heart disease requires accurate knowledge of cardiac ultrastructure, protein distribution and subcellular function. As current imaging techniques are limited in spatial resolution, limiting insight into changes in calcium handling, computational models of excitation–contraction coupling have been increasingly employed to probe these structure–function relationships. This review will focus on the development of structural models of cardiac calcium dynamics at the subcellular level, orienting the reader broadly towards the development of models of subcellular calcium handling in cardiomyocytes. Specific focus will be given to progress in recent years in terms of multi-scale modeling employing resolved spatial models of subcellular calcium machinery. A review of the state-of-the-art will be followed by a review of emergent insights into calcium-dependent etiologies in heart disease and, finally, we will offer a perspective on future directions for related computational modeling and simulation efforts.

## Introduction

Excitation–contraction coupling (ECC) in cardiomyocytes requires calcium (Ca) influx through L-type Ca channels (LCCs) in the sarcolemma, which initiates Ca release through ryanodine receptors (RyRs) clustered in the terminal cisternae of the sarcoplasmic reticulum (called junctional SR [jSR]) in a process known as Ca-induced Ca release (CICR). Ca influx via an a intracellular Ca transient (CaT), enabling cardiomyocyte contraction. Ca is removed from the myoplasm, ending the CaT, via the sarco/endoplasmic reticulum Ca-ATPase (SERCA) and by the sarcolemmal Na–Ca exchanger (NCX) as well as the Ca pump (CaP). All RyRs and associated jSR structures that can be activated as a distinct unit are denominated the “calcium release unit” (CRU).

**Figure 1.**
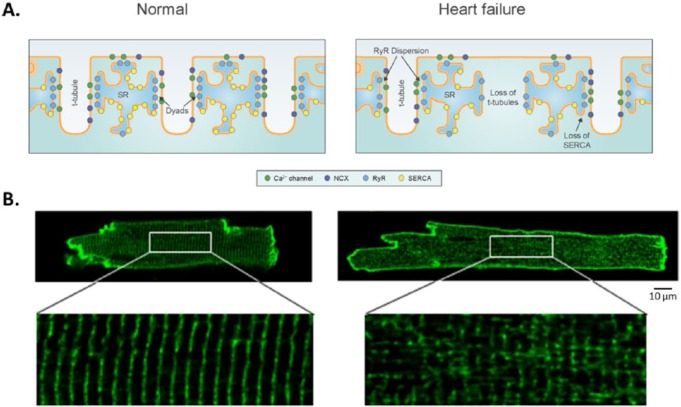
CRU organization and t-tubular structure in the normal (left) and failing (right) cardiac myocyte. In healthy ventricular cardiac myocytes, L-type Ca channels directly appose RyRs in each CRU, and t-tubular structure is regular (A and B, left column). However, in the setting of heart failure, disruption of the t-tubular network (B, right column) causes RyR dispersion (A, right column), leading to abnormal calcium transients. Disruption of the t-tubule network (B, right column) and RyR dispersion (A, right colum) leads to abnormal calcium transients. (Adapted from Louch et al.,^38^ used with permission.)

The spatio-temporal dynamics of CICR, buffering, and reuptake into the SR play a central role in ECC in both normal and diseased cardiac myocytes. In cardiac myocytes, it has been proposed that 5–15 LCCs embedded in the sarcolemma appose 50–200 clustered RyRs as distinct structures^1^ (see Figure 1A, left panel). However, the exact numbers and ratio of LCCs and RyRs in these functional couplons is an area of ongoing research. The dyad is considered to be a single-sided lobe of the jSR apposing the transverse-tubule (t-tubule) membrane, invaginations of the sarcolemma of cardiomyocytes. The dyadic geometry estimated to have a radius of 0.05–0.2 *µ*m, and a height of 10–12 nm, can alter in disease and displays significant interspecies variability.^2^ Several characteristic properties of ECC, such as high gain and graded Ca release, arise from interactions that occur in and between these local dyadic microdomains. Dyads are clustered along t-tubules. Mammalian ventricular cells typically have a well-developed, regular structure for t-tubules (t-network). Atrial cardiomyocytes from large mammals have been shown to have well-developed t-tubular networks; however, species differences and specifically a lack of defined t-tubular structure in atria myocytes from small mammals has historically led to atrial t-tubules being overlooked. The t-tubular system plays a central role in the synchronization of Ca signaling and ECC in many striated muscle cells; disruption of the t-network contributes to dyssynchronous Ca release and impaired contraction.^3–7^ CICR in small dyads gives rise to high gain through positive feedback (an all or none event), but the spatial distribution and relative isolation of CRUs allows for sequential recruitment and graded release. The restricted number of molecules in each CRU can mean that approximating dynamics as continuous is inappropriate: processes therein may be better described by stochastic as opposed to deterministic models. Many earlier models of the cardiac action potential did not include descriptions of CICR that accounted for these local mechanisms.^8^

Ca signaling in the CRU is a fundamentally discrete process;^9^ short-lived, local increases in intracellular Ca via triggered SR release are known as Ca sparks, which regulate the generation of whole-cell CaT and ECC. For example, spontaneous Ca sparks have durations of 10–100 ms, allowing SR Ca uptake to keep pace with release, a process which cannot happen during a complete triggered release event. Long-lasting sparks with durations of several hundred milliseconds to seconds, so-called “embers,” are also widely observed. Experiments have shown that the transition from normal to long-lasting sparks can occur when RyRs open probability is reduced.^10^ Dysfunction in Ca handling is central to a number of cardiac pathologies (including heart failure (HF) and atrial fibrillation (AF)) and may lead to mechanical dysfunction as well as arrhythmia. Disruption of dyadic structure is thought to be largely responsible for changes in Ca handling; we will delve further into these aspects in later sections of this review. Further quantitative understanding of cardiomyocytes’ Ca machinery and the study of mechanisms that underlie both normal cardiac function and calcium-dependent etiologies in heart disease thus requires accurate knowledge of cardiac ultrastructure, protein distribution and subcellular function. As current imaging techniques are limited in the spatial resolution to which changes in Ca can be detected, computational models of ECC have thus been increasingly employed to probe these structure–function relationships. Mathematical modeling of ECC in cardiomyocytes is a fundamentally multi-scale problem: it involves gradients on the spatial scale of 100 nm or even less in dyadic clefts and concentration profiles along the 100 *µ*m of the whole cell, as well as the submillisecond timescale of local concentration changes and the change of SR Ca content within tens of seconds.

This review will focus on the development of structural mathematical models of cardiac Ca dynamics. While of importance for select cardiac pathologies, in general the role of mutation and protein dysfunction or dysregulation will not be treated in detail here, nor will the study of targets or putative drug therapies for Ca handling dysfunction take a major role. Discussion of mitochondrial electrodynamics and signaling, though offering critical insight, will not be treated here and have been reviewed elsewhere,^11^ nor will the explicit roles of oxidative stress or CaMKII signaling.^12,13^ Others have also offered excellent reviews of modeling of myocardial Ca at different spatial scales from the perspective of parameter sensitivity analysis,^14^ which will not be the primary focus at present. Instead, the present review will orient the reader broadly towards the development of models of subcellular Ca handling in cardiomyocytes. We will place specific focus on progress in recent years in terms of multi-scale modeling employing resolved spatial models of subcellular calcium machinery. A review of the state-of-the-art will be followed by a review of emergent insights into calcium-dependent etiologies in heart disease and, finally, will offer a perspective on future directions for related computational modeling and simulation efforts.

## State-of-the-art: established and emerging methods

Mathematical modeling of the physiology of both atrial and ventricular cardiomyocytes is a broad field including several mathematical formalisms. For the convenience of the less-familiar reader, we here offer a brief review of a few common computational modeling terms used in the present review. Something which is discretely valued is constant over a spatial or temporal interval, while one that is continuous would have smoothly varying values in time and space; binary in this context refers to an all-or-nothing phenomenon. A compartment refers to a restricted virtual space in the mathematical model which represents a volume considered to be separate from a common or bulk space in the interior of the cell. A deterministic model refers to a system wherein no randomness is involved in calculation of its future states. A deterministic model will thus always produce the same output from a given input, while stochasticity refers to event or system that includes uncertainty in outputs because of random variation in one or more inputs over time. Markov processes are stochastic processes that satisty the property of “memorylessness;” the concept is that one can make predictions for the future based only on the present state, with the future and past as independent. Monte Carlo simulations are used to model the probability of different outcomes in processes that cannot easily be predicted because of stochastic inputs. In a single trial, a range of values (probability distribution) is substituted for a number for any factor that has inherent uncertainty; with many, many trials, each using a different set of random values, a complete picture of probable outcomes is obtained. The probability density function (arising from statistics) is a function used to specify the probability of a random variable falling within a particular range of values, as opposed to taking on any one value.

### Models of subcellular calcium handling: an overview

**Figure 2.**
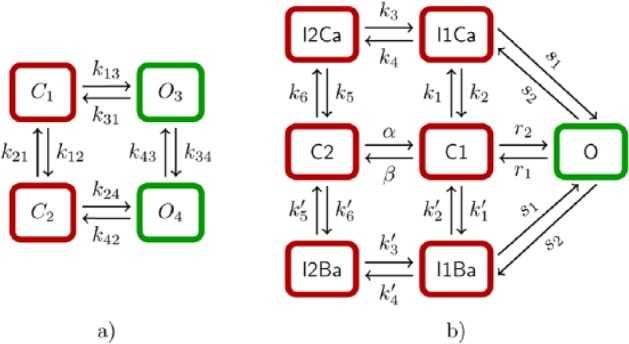
(a) A four-state Markov model for ryanodine receptors (RyRs). If the horizontal and vertical transitions are independent of each other (*k*_13_ = *k*_24_, *k*_31_ = *k*_42_ and *k*_12_ = *k*_34_; *k*_21_ = *k*_43_), then the model can be expressed with the Hodgkin–Huxley formalism. (b) A seven-state Markov model for L-type Ca channels (LCCs), from Mahajan et al.^92^

While models of ECC in cardiomyocytes developed over the past 30 years have been reviewed completely elsewhere,^15,16^ a brief orientation follows. Movement and dynamics of Ca ions in the dyad have often been described by assigning continuously valued Ca concentrations to one or more dyadic compartments; several models have been based upon deterministic representations (without stochasticity) wherein CRUs were lumped into a single “common pool.”^17–19^ As the RyRs in this common pool are activated via influx of Ca through LCCs, the strong positive feedback of CICR ensures that the pooled release units activate completely, resulting in coordinated, binary calcium release. Ventricular cardiomyocytes, however, display a graded response to trigger Ca in vivo: as the amount of trigger Ca from LCCs goes up, so does release via the SR through the RyRs. While some of the deterministic computational models do simulate graded release, these require an artificial mechanism to do so.^8^ Stern^20^ demonstrated that high-gain graded release could not be simulated via common-pool means, but that modeling CRU including local CICR triggers and recruitment of a neighborhood cluster of RyRs permitted graded response; this was also later demonstrated in a more physiological model.^21^ Thus, a sufficient increase in dyadic subspace Ca following local LCC opening causes apposed RyRs to open; specifically, then, stochastic recruitment of neighboring CRU results in graded release: the so-called “local control”-type model. These stochastic properties of the system can be reduced to a representative Markov model based on principles of timescale decomposition. The RyRs and LCCs are often modeled via Markov state models, as reviewed elsewhere^16^ (see Figure 2 for examples of four- and seven-state models, respectively).

Local control models of Ca release wherein interactions between LCCs and RyRs are simulated stochastically are able to reconstruct the key physiological phenomena of both graded Ca release and high CICR gain,^22–25^ but at a high computational cost relative to common-pool models. Approaches have sought to reduce the cost levied by stochastic simulation; a breakthrough model presented a general analytical approach for deriving simplified models of local control of CICR.^26^ The resulting coupled LCC–RyR gating model successfully reproduced the LCC response to voltage-clamp stimuli, and the inactivation of LCCs with and without Ca release from the SR found in experiments, at reduced computational cost. In another non-spatially resolved model, a probability density function approach was used to replace the costly Monte Carlo simulations necessary to model local control via stochastic Markov processes. The method derived coupled advection–reaction equations relating the time-dependent probability density of subsarcolemmal subspace and junctional SR Ca concentration ([Ca]) conditioned on the CRU state. Modeling CRU activity using this probability density approach avoids resolution of precise spatial aspects of global Ca signaling, but represents heterogeneous local Ca signals in a population of dyadic subspaces and junctional SR depletion domains with reasonable accuracy.^27^ Both approaches to modeling local control of ECC produce high-gain Ca release that is graded with changes in membrane potential, phenomena not exhibited by common pool models. Other non-spatially resolved models of local control have linked the processes of subcellular Ca machinery to whole cell behavior.^28,29^

### Pseudo-spatial and spatially-resolved models of subcellular calcium processes

The local nature of the ECC control mechanism was not clear until the discovery of Ca sparks by Cheng et al.^30^ In recent decades, there has been much additional evidence that the control of CICR is contingent upon local Ca in the immediate vicinity of the CRU, rather than on the whole-cell Ca.^31,32^ This tight, local regulation of CICR is made possible by the clustering of LCCs and RyRs into discrete couplons, rendering them sensitive to local rather than global Ca. While Ca sparks represent the “unit” of Ca release from large arrays of RyRs in the CRU, Ca “blinks” represent the Ca depletion signal produced in the terminal cisternae of the jSR. Furthermore, so-called quarky Ca release observes release events smaller than sparks at a level substructural to the CRU.^33^ In other words, macroscopic Ca release events are intrinsically controlled by the type and number of individual LCCs and RyRs in the CRU, the relative spatial localization of the two channel types and geometry of this functionally significant nanodomain, as well as the organization of the CRUs at the cell level.

An early model of Sobie et al.^23^ consisted of a single LCC closely apposed to a cluster of RyRs in a region of jSR. While simple, the stochastic current descriptions for RyRs and LCCs sought to lend insight to the question of Ca spark termination via the novel “sticky cluster” model, meaning a model that incorporated the “sticky” cooperativity between adjacent RyRs. Next efforts employed explicitly spatially-resolved single CRUs; one study to investigate the effects of action potential prolongation in a model of murine HF used a rectangular space with dimensions 100 nm × 100 nm and a dyadic cleft space of 12 nm between the t-tubular and SR membranes, analysis revealed that Ca spark amplitude and rise time were highly dependent on the number of activated channels (LCCs and RyRs) and their packing within the CRU, though not very sensitive to other cleft dimensions.^34^ More detailed was a study by our groups which developed a three-dimensional computational model of a single dyad, modeled as a cylindrical disk,^35,36^ with CRUs of two sizes: 25 or 100 RyRs per CRU, with RyRs positioned in a highly regular two-dimensional lattice grid.^6^ In a follow-up study on whether RyR function promoted slowing of Ca release in murine HF, the sticky cluster model was re-parameterized^37^ and, rather than incorporate a highly regular RyR geometry in the CRU, was extended to include subclusters in a CRU in addition to one “mother cluster.” All subclusters were connected with the mother cluster through diffusion but not with each other, and Ca sparks were initiated by opening one RyR in the mother cluster.^38^

Computational models have additionally moved towards the level of the whole myocyte, generally stepping up dimension and complexity in geometry. Tao et al.^39^ used a schematic model of a cluster of coupled RyRs in a cardiac ventricular cell of length of 150 *µ*m, with 2 *µ*m spatial resolution^40^ to study intracellular Ca alternans (see Section 3.3) by coupling model elements via Ca diffusion between neighboring cytoplasmic and network SR spaces. The more complex model of Rovetti et al.^41^ developed a quasi-two-dimensional spatially-distributed Ca cycling model via network of 100 by 100 CRU which included a network SR (nSR) domain and a myoplasmic domain coupled via SR Ca release and uptake. The model comprises a CRU network coupled via Ca diffusion in each domain. Each CRU contained a jSR diffusively connected to the nSR, and a dyadic space diffusively connected to the myoplasm, as well as stochastic LCCs (5 channels per CRU) and RyRs (100 channels per CRU). Gaur et al.^42^ developed an initial multi-scale model of a spatially distributed mammalian ventricular myocyte consisting of 10,000 diffusively coupled CRU, with the number of stochastic LCCs and RyRs in a dyad as 15 and 100, respectively, to investigate how microscopic changes in dyadic properties including detubulation in HF, can affect whole-cell behavior. A recent model of ECC in the mouse cardiac ventricular myocyte, developed to further elucidate the physiologic consequences of leaky RyRs, included a three-dimensional spatial implementation of the same group’s local control model, representing a single sarcomere centered on a z-line and containing equally distributed CRU (inter-CRU distance is 600 nm).^43^ Cannell and Laver^44,45^ have recently employed with others a cylindrical model of the dyad with a t-tubule at the center to investigate control and termination of CICR via the SR; importantly, this model permits calcium gradients within the CRU.

Others have used similar approaches to examine local Ca in the atrial myocyte. The observation of variable t-tubule density in atrial myocytes^46^ has not yet been taken into account in several models; instead, models have assumed wave-like CICR propagation of Ca into the cell interior. Given cell-type potential differences in ultrastructure, the “z-plane” distribution of RyRs was modeled radially, rather as a regular grid in one study, to monitor the three-dimensional diffusion of Ca along a portion of the cell.^47^ Another model of the human atrial cardiomyocyte^48^ included a spatial representation of Ca-handling based on longitudinal division into ~2*µ*m wide segments, and in the transverse division into ~1*µ*m long domains, as based on the model of Grandi et al.^49^

**Figure 3.**
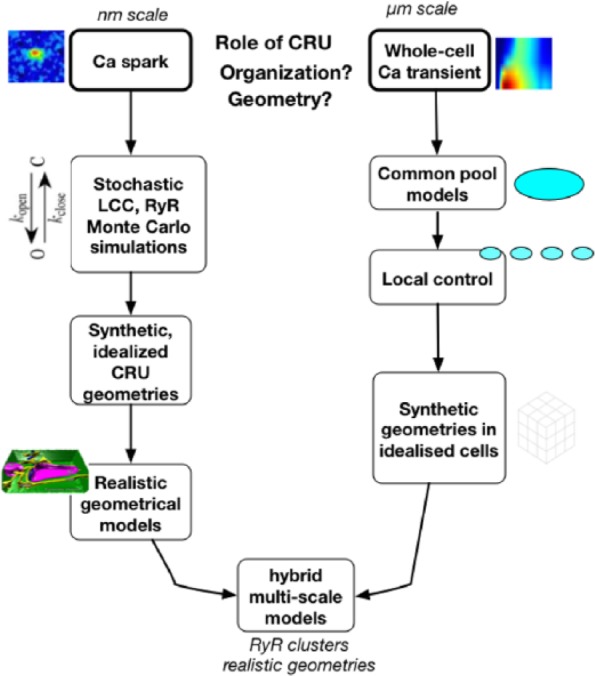
An overview of the development of spatially resolved models of the cardiac myocyte. Modeling of subcellular processes emerged from the need to understand the link between the Ca spark, calcium release unit (CRU) organization and geometry, and whole-cell emergent behavior. Modeling of Ca sparks at the level of the CRU have employed stochastic ryanodine receptors (RyRs) and L-type Ca channels (LCCs) and Monte Carlo simulation in first synthetic and idealized, and, more recently, real geometries, to offer insight into spark dynamics, including spark termination. Concomitantly, whole-cell calcium dynamics modeling began with common-pool representations, replaced by local-control models and sticky-cluster modeling including the effects of RyR cooperativity on Ca release. These models have been stacked into repeating units to approximate whole-cell function, first with generic geometries in idealized cells, to reconcile observed phenomena such as graded release with high gain and cellular alternans. Incompletely resolved questions include how much LCC current is actually required for RyR activation, the phenomena of long-lasting “embers,” and how CRU structure collectively impacts waves, subtle aspects of CRU recruitment and modifies the stability of the dynamical range in which the CRU/couplon ensemble is operating. (Image reproduced from Hake et al.^58^ with permission.)

Physiologically detailed models of subcellular Ca cycling including a three-dimensional network of ~20,000 CRU at the level of the whole ventricular cell have been developed by Restrepo and colleagues.^50–54^ The model and its iterations include a three-dimensional network of 19,305 (65 × 27 × 11) CRUs with CRU spacing at 1.84 *µ*m (longitudinal) and 0.9 *µ*m (transverse direction), corresponding to a ventricular cell of dimension ~ 120 × 25 × 10 μm^3^. CRUs are coupled via Ca diffusion in the cytosolic space and SR. Each CRU contains five subvolumes: the nSR, jSR, dyadic space or proximal space, a submembrane space, and a cytosolic space. While spatially resolved, each CRU incorporates a cluster of 100 RyR channels and 10 LCCs, both simulated using random Markov transitions: cluster size is fixed, other aspects of CRU geometry not taken into account, and the CRU is considered a common-pool with respect to [Ca] gradients. Others have used a similar approach, but simplified the three-dimensional problem to a two-dimensional model of a cardiac myocyte,^55^ similar to the work by Izu et al.^56^ Recently, Sato et al.^57^ expanded upon the work of Restrepo et al. with RyR clusters of a few to several hundred RyRs, varying the number of functional RyRs in a single cluster, diffusion within the SR network, diffusion between network and junctional SR, cytosolic Ca diffusion, SERCA uptake activity, and RyR open probability. To address the need for explicit representation of CRU geometry, Hake et al.^58^ developed novel tools to generate computational geometry from electron tomographic images and created a detailed computational model of a single CRU. Ca diffusion was modeled within the SR and the cytosol to examine the effects of localization and density of the NCX, SERCA, and CSQN. Others have developed high-resolution imaging and analysis approaches to measure the three-dimensional distribution of immunolabeled proteins with confocal microscopy in cardiomyocytes as both RyRs and t-tubular system distributions show large variation from the simple grid geometries assumed in previous work; this three-dimensional RyR cluster distribution has been used to construct a model of stochastic Ca dynamics in a myocyte.^59^ Figure 3 offers an overview of the development of models of pseudo-spatial and spatially-resolved models of subcellular calcium release.

### Multi-scale modeling of calcium-driven processes in cardiac electrophysiology

Computational models at present seek to combine the data emergent from newer imaging technologies to gain multi-scale insight into calcium-driven processes. Spatial point process statistics techniques were recently developed to simulate the spatial distribution of RyR clusters, combining confocal-scale (~200 nm) data of RyR clusters with three-dimensional electron microscopy data (~30 nm) of myofibrils and mitochondria, both collected from adult rat left ventricular myocytes. This hybrid-scale spatial model was employed in reaction–diffusion Ca simulations during the rising phase of the CaT.^60^ Another recent study introduced a new concept for a multi-scale mathematical model of CICR and whole cardiomyocyte electrophysiology. This incorporated stochastic simulation of individual LCCs and RyRs, spatially detailed concentration dynamics in dyadic clefts, rabbit membrane potential dynamics, and a system of partial differential equations (PDEs) for intracellular and SR free Ca as well as Ca-buffering, and resolved concentration gradients from the level of the dyad to the whole-cell level by using a quasi-static approximation in CRU.^61^

## Emergent results: insights into Ca-handling mechanisms

### Mechanistic insight into normal function

Multi-scale computational models of subcellular Ca handling have offered a wealth of mechanistic insight into the cardiomyocytes’ function in the normal heart. To illustrate, models of stochastic molecular signaling between LCCs and RyRs describing known features of dyad geometry, including key dyadic proteins and electrodiffusive movement of individual Ca ions enabled investigation of how local Ca signaling is influenced by the dyad structure. The geometry of the individual RyR may indeed function to restrict the diffusion of and to “funnel” Ca ions to activation-binding sites on the RyRs, increasing RyR open probability and ECC gain.^24,62^ Furthermore, models incorporating realistic CRU distributions permitted Ca waves that spread axially along the cell at observed velocities, demonstrating that spatial features of the CRU distribution on multiple length scales critically affects intracellular Ca dynamics.^59^ A recent study showed that RyRs positioned axially helped propagate Ca waves in the axial direction.^63^ Greenstein et al.^29^ generalized an earlier analytical approach for deriving simplified mechanistic models of CICR to formulate an integrative model of the canine cardiac myocyte, which was used to study the role of local redundancy in LCC channel gating and the role of dyad configuration on ECC. Simulations suggested that the characteristic steep rise in ECC gain observed at hyperpolarized potentials is a result of increased functional coupling between LCCs and RyRs.

### Calcium sparks and calcium waves

Modeling and simulation has permitted detailed insight into Ca spark generation in normal function. The Williams et al. model for calcium dynamics in the rat ventricular myocyte includes 20,000 CRU consisting of clusters of stochastically gated RyRs.^64^ The model resolves the multiple single opening events that do not result in a Ca spark (~3,000 per cell per second), and also shows that a single RyR release event can trigger others via CICR and that once ~12% of RyRs are open, a spark usually follows. The termination of a calcium spark has been an active research topic for many years with several hypotheses proposed; the reader is referred to an excellent recent review.^65^ The spatial arrangement of RyRs within clusters has a major influence on the frequency of Ca sparks. The probability of a Ca spark occurring when a single RyR in the cluster opens spontaneously can be predicted from the precise spatial arrangement of the RyRs; function follows directly from structure, in this case. A computational model of the dyadic cleft which specified the spatial localization of LCCs and RyRs revealed that reaction specificity and efficiency are regulated by microdomain geometry as well as the physical separation of signaling molecules into functional complexes. Both Ca spark amplitude and rise time were found to be highly dependent on the concentration of activated channels in the CRU microdomain and on the intermembrane separation channel packing (Koh, 2006).^34^ Previous model studies also have predicted that the duration of the spark is determined by the local CRU geometry, as well as the localization and density of Ca-handling proteins.^58^ It has been newly demonstrated that long-lasting Ca sparks emerge as a collective dynamical behavior of a network of diffusively coupled CRU; there exists an optimal range of RyR open probability favoring long-lasting sparks.^54^ Another recent study employed a spatially resolved mathematical model of subcellular Ca cycling to examine how Ca spark duration is influenced by the number of functional RyRs in a junctional cluster and other SR Ca-handling properties; if the number of RyRs is under a certain threshold, it is difficult to maintain consecutive openings and stochastic attrition terminates Ca release while, if the number of RyRs in a cluster is too large, the depletion of Ca from the jSR terminates release. It was found that protracted Ca release events require small RyR clusters and sufficiently rapid intra-SR Ca diffusion.^57^

Intracellular Ca waves are a form of Ca signaling executed in many cell types and can occur in cardiac myocytes during Ca overload.^30^ Spontaneous increase in [Ca] can occur at a single or multiple CRU within a cell and can lead to propagation throughout the myoplasm in a wave-like pattern. Cheng et al.^30,66^ speculated that Ca waves arose from the collective firing of Ca sparks; that Ca released in a dyad can diffuse and trigger Ca release in adjacent dyads, forming traveling waves. The nature of this propagating mechanism means that a wave travels at a finite velocity. This is therefore dissimilar to the CaT evoked by an action potential, which is a whole-cell release event, coordinated by depolarization-activated Ca entry through LCCs.^67^ Ca waves are a natural consequence of CICR; the evolution of Ca wave models reflects the growth of knowledge of ECC in muscle. The appearance of Ca waves may lead to whole-cell depolarization and triggering of an action potential, as these may activate inward currents such as that carried by the NCX.^68^ Thus, there exists a putative link between Ca waves and triggered activity leading to initiation of dangerous arrhythmias; experimental studies have demonstrated that abnormal Ca cycling is a critical factor in the development of focal excitations.^69^ Spatially-resolved cell models have shown that the time needed to form cluster of sufficient size to elicit a Ca wave, as well as the critical cluster size, becomes smaller as SR Ca load and diastolic myoplasmic Ca increases.^52^ A separate study in a similar model effected sensitivity analyses to study physiological parameter effects on global Ca waves: computed results were in agreement with confocal microscopy imaging, and found that the current flow amplitude through the CRU affected dynamic properties of Ca waves more significantly than the duration of this current, and that longitudinal and transverse separation of CRU affected the longitudinal velocity and amplitude of Ca waves significantly.^55^

### Calcium alternans

Cellular CaT alternans are beat-to-beat alternations in the peak cytosolic calcium concentration exhibited by cardiac cells during rapid electrical stimulation or under pathological conditions. CaT alternans promote action potential duration alternans, which have been linked to the onset of life-threatening ventricular arrhythmias. Ca alternans in cardiac myocytes have been shown in many experimental studies, and the mechanisms remain incompletely understood. The ability to link microscopic properties of CRU to whole cell behavior is thus a powerful tool to investigate the arrhythmogenic role of abnormal Ca dynamics in cardiac disease. A study employing the multi-scale model of Restrepo et al.^50^ showed that luminal (SR-side) gating of the RyRs mediated by CSQN can cause calcium transient alternans regardless of the steepness of the release–load relationship: alternans were caused by a beat-to-beat alternation in the number of refractory RyR channels and could occur with or without diastolic SR calcium content alternans. The same group showed that ion channel stochasticity at the level of a single CRU can influence the whole-cell alternans. Depending on the sign and magnitude of Ca–voltage coupling, Ca alternans can be spatially synchronized or desynchronized, and in or out of phase with action potential duration alternans. Calcium alternans can, for instance, be spatially synchronized but out of phase with action potential duration alternans.^51^ The concurrent model of Tao et al.^39^ found that alternans of systolic Ca were generated by propagating Ca waves sustained through alternation of SR Ca content, implicating additional mechanisms for intracellular Ca alternans in addition to refractoriness of LCCs or RyRs under rapid pacing. Rovetti et al.^41^ showed that Ca alternans emerge as a collective behavior of Ca sparks, determined by the CRU network, via three Rs: “randomness” (of Ca spark activation), “refractoriness” (of a CRU after a Ca spark), and “recruitment” (Ca sparks inducing Ca sparks in adjacent CRUs). Nivala et al.^53^ later employed the ventricular myocyte couplon network model to study how SR Ca load and other physiological parameters, such as RyR sensitivity, SR uptake rate, NCX current, and Ca buffering affect Ca alternans in the context of the 3R theory, and found that alternans only occurs for an intermediate range of the SR Ca load, and the underlying mechanism can be explained via its effects on the three Rs (randomness, recruitment, and refractoriness).

## Understanding disease: insights into mechanisms

### Atrial fibrillation and atrial arrhythmia

Electrical, structural, and Ca handling remodeling contribute to the perpetuation and progression of AF. Evidence has suggested a role for Ca leak and spontaneous SR Ca release events at several stages of disease progression,^70–73^ and arrhythmogenic Ca waves resulting from heterogeneities in subcellular Ca alternans have been implicated as a mechanism in atrial dysrhythmia.^74^ Studying structure–function relationships and aberrant calcium handling in AF is complicated, however, by the fact that there are significant species-based differences in ultrastructure; whereas many large mammals, including humans, evince t-tubules in atrial cells,^75,76^ smaller mammals may not, and regional differences introduce further heterogeneity. The potential import of the t-tubular network in large mammal atrial EC coupling, despite the likely profound importance in subcellular Ca dynamics in these cells, has largely been overlooked thus far, and is an active subject in emerging modeling approaches at date of publication. Some human atrial myocytes do lack t-tubules (and thus compose an easily discretized structure as a starting point) and contain SR of both the junctional and non-junctional types, both of which have RyRs. An innovative mathematical modeling approach allowing detailed characterization of Ca movement within the three-dimensional volume of an atrial myocyte, displaying the centripetal Ca waves that occur within atrial myocytes during EC coupling and demonstrated that altering the strength of Ca release, RyR refractoriness, the magnitude of initiating stimulus, or the introduction of stochastic Ca channel activity could cause the nucleation of proarrhythmic traveling Ca waves.^47^ An influential combined experimental-computational study in human atrial cardiomyocytes from patients in sinus rhythm or with paroxysmal AF (pAF) showed increases in SR Ca leak and incidence of delayed after-depolarizations in pAF, underpinned by increased inactivation (phosphorylation) of the SERCA inhibitor protein phospholamban in pAF (thus, increased SERCA function), and increased RyR expression and single-channel open probability. Computational modeling indicated that both RyR dysregulation and enhanced SERCA promoted SR Ca leak and spontaneous SR Ca-release events, causing delayed afterdepolarization and potential triggered activity in pAF.^48^ In addition, while Torres et al.^77^ developed a model including the spatial arrangement of the sarcolemma including t-tubular system in ventricular myocytes, their findings were explained by a modified local control model, which constrained the region of regenerative activation of non-junctional RyR clusters, which may prove useful for describing ECC in AF cardiac myocytes, with a sparse t-system More recent work into RyR cluster fragmentation and redistribution in persistent AF^78^ motivate the further use of computational models to elucidate the role of structural disturbance in atrial dysrhythmia.

### Heart failure

The abnormalities in Ca handling occur at nearly every point of Ca cycling in the failing heart cell, including activation and termination of SR Ca release, diastolic SR Ca leak, and SR Ca uptake.^79^ Cardiomyocytes from failing hearts exhibit a characteristic slowing of the rising phase of the CaT and additionally exhibit spatially nonuniform or dyssynchronous SR Ca release. A combined experimental/computational study from our groups used a computational model of the dyad to investigate the contribution of AP prolongation in a murine model of HF; ultimately, the study found that dyssynchronous Ca release in HF mouse myocytes does not result from electrical remodeling, but rather other factors, such as t-tubule reorganization (Figure 1B, right panel),^6^ as the longer murine action potential in HF resulted in increase SR Ca content, offsetting the desynchronizing effect of the extended action potential in this species. Furthermore, related work established that synchrony of cardiomyocyte Ca release is not only determined by t-tubule organization but also by the interplay between RyR sensitivity and SR Ca content. Cardiomyocytes from failing hearts also exhibit slow, dyssynchronous CaTs resulting from a subset of Ca sparks with slow kinetics.^38^ Slow sparks may occur at intact dyads: slow sparks are predicted to result from reorganization of CRUs in HF (Figure 1A, right panel). In addition to impaired contraction, this aberrant intracellular Ca cycling in HF has been implicated in both triggered and reentrant arrhythmias.^80^ The model of Gaur and Rudy^42^ was used to investigate how changes in microscopic dyadic properties, including detubulation in HF, affect whole-cell behavior. They found that increased dyadic volume and reduced LCCs/RyRs decrease ECC gain and can cause asynchrony of SR Ca release; when CSQN function is decreased, interdyad coupling increases diastolic Ca release activity to form Ca waves and long-lasting Ca release events. A recent study using a spatially-resolved ventricular myocyte model (see section 2) investigated effects of t-tubule disruption and other HF remodeling factors (CRU refractoriness, CRU coupling, and RyR leakiness) on Ca alternans. While others have seen that detubulation reduces the likelihood of sparks,^6^ in this model, disruption removed LCCs from the associated CRUs, and resulted in orphaned RyR clusters, providing increased opportunity for spark-induced Ca sparks to occur.^2^ The authors found that this t-tubular disruption promoted Ca alternans by two distinct mechanisms (1) (with normal SERCA function) by both CRU refractoriness and inter-CRU coupling, and (2) in the context of down-regulated SERCA, alternans was caused by an SR Ca load-dependent mechanism, independent of CRU refractoriness. The authors concluded that the mechanisms of Ca alternans for normal and down-regulated SERCA are different, and that t-tubular disruption promotes Ca alternans by both mechanisms, which may contribute to alternans at different stages of HF.^81^

## Future directions: continued advances in imaging

Multi-scale meshes are key for computational studies of structure–function relationships in ECC. An early study aimed at developing an approach for spatial reconstruction of structures involved in calcium handling reconstructed clusters of RyRs together with the sarcolemma as based on dual labeling and three-dimensional confocal imaging of myocytes, leading to three-dimensional stacks of cross-sections; digital image processing was applied to deconvolve, filter, and segment image stacks.^82^ Clearly, advancing computational models via spatially resolved approaches now relies in part upon advances in experimental and imaging technologies which permit leaps in computational models.^83^

Advances in microscopic imaging technologies such as serial block face scanning electron microscopy (SBF-SEM) now allow description of new micro-domain in cardiomyocytes.^84,85^ Three-dimensional electron microscopy technologies such as electron tomography have been able to determine realistic nanogeometries of membrane junctions (dyads and peripheral junctions) and associated t-tubules. Labeling with antibodies has allowed examination of the three-dimensional distribution of RyRs with confocal microscopy,^86^ revealing couplon geometries later used in detailed computational models.^44,45^ At the same time, super-resolution light microscopy has gone beyond the diffraction limit to determine the distribution of smaller dyadic molecules, such as LCCs, at unprecedented resolutions, offering insight into the central machinery controlling cardiac ECC via calcium signaling.^87^ Computational models built upon such technologies are able to furnish unprecedented insight.^58^

**Figure 4.**
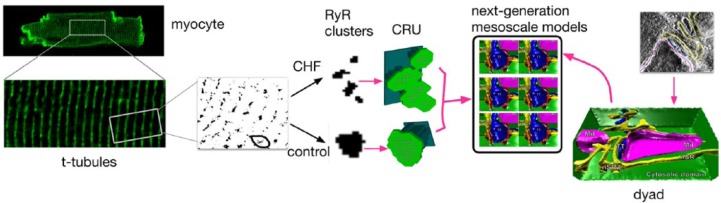
Representation of the next generation of subcellular computational models, from left: super-resolution light microscopy permits resolution of the morphology of ryanodine receptor (RyR) clusters, which can be incorporated into synthetic geometries of the calcium release unit (CRU). Using these synthetic geometries, one can easily and systematically alter the distribution of RyRs, the shape and volume of the junctional SR (jSR) and network SR (nSR), and the cleft volume, and begin to analyze the different contributions quantitatively, permitting query into how spark fidelity is affected by RyR density, by cluster breakup, by cleft height, or by small and narrow jSR (local depletion of Ca). On the other hand (from right), these synthetic geometries neglect the potentially important role played by detailed and realistic CRU structures, which can now be obtained from electron tomography. (Images reproduced from Hake et al.^58^ with permission.)

Correlated light and electron microscopic (CLEM) imaging is a powerful method wherein each imaging mode provides unique information for dissecting cell and tissue function at high resolution.^88^ Other recent approaches have combined fluorescence resonance energy transfer (FRET), simulated-annealing (a form of combinatorial optimization), cryo-electron microscopy, and crystallographic data to locate a biosensor peptide bound to RyR Ca channels^89^ and have targeted a new sensitive Ca biosensor to the junctional space, where it co-localized with t-tubules and RyRs, allowing selective visualization and measurement of nanodomain Ca dynamics in intact cells.^90^ These multi-scale experimental and imaging approaches will offer mechanistic insights into CRU RyR operations in health and in disease states, and additionally offer potential for future inclusion in mechanistic computational modeling. Furthermore, emerging super-resolution single-molecule localization microscopy (SMLM) techniques offer an order of magnitude improvement over resolution of conventional fluorescence light microscopy; nanometer-scale distributions of multiple molecular targets can be resolved. In conjunction with the next generation of electron microscopy, SMLM has allowed the visualization and quantification of intricate t-tubule morphologies within large areas of muscle cells at unprecedented levels of detail, as recently reviewed.^91^ Novel and emerging imaging methods will enable the incorporation of detailed subcellular structural and functional information into the next generation of computational models (Figure 4), providing entirely new insights into the ion dynamics underpinning excitation and contraction in the heart, as well as the ways in which the system can fail in cardiac disease.

## Summary and conclusions

In the present review, we have focused on the development of structural models of cardiac Ca dynamics, introducing the reader to the development of models of subcellular Ca handling in cardiomyocytes, with specific focus on progress in recent years. Computational modeling and simulation have helped to uncover the extent to which macroscopic Ca release events are intrinsically controlled by the type and number of individual LCCs and RyRs in the CRU, the relative spatial localization of the two channel types and the geometry of this functionally significant nanodomain, as well as the role of CRU organization at the cell level.

Computational models have additionally moved towards the level of the whole myocyte, generally stepping up dimension and complexity in geometry. Both subcellular models investigating spark dynamics at the level of the single CRU and physiologically detailed models of whole-cell subcellular Ca cycling including networks thousands of CRU have been developed, at present seeking to combine the data emergent from newer imaging technologies to gain multi-scale insight into calcium-driven processes. This research has offered new knowledge into Ca spark termination, and, most recently, long-lasting Ca sparks. Structural distribution of CRU have additionally been shown to affect dynamics of Ca waves. Biophysical models have furthermore been employed to show that Ca alternans emerges as a collective behavior of Ca sparks, determined by the CRU network, via randomness of Ca spark activation, refractoriness of a CRU after a Ca spark, and recruitment, inducing Ca sparks in adjacent CRUs. Other computational strategies have offered new insight in the context of AF, wherein research revealed the role of RyR cluster fragmentation and redistribution on Ca remodeling in persistent AF, as well as HF, where the role of t-tubular disruption on electrical abnormalities (alternans) has been studied.

Finally, novel labeling and imaging techniques now permit selective visualization at the nanodomain in intact cardiac myocytes, offering needed intelligence into Ca operations in health and disease, as well as a platform for the next generation of mechanistic computational models. These emerging multi-modality experimental methods will enable incorporation of detailed subcellular structural as well as functional information, providing powerful new computational tools for insight into the dynamics underpinning excitation and contraction in the heart.
